# α-Ketol and α-iminol rearrangements in synthetic organic and biosynthetic reactions

**DOI:** 10.3762/bjoc.17.172

**Published:** 2021-10-15

**Authors:** Scott Benz, Andrew S Murkin

**Affiliations:** 1 Department of Chemistry, University at Buffalo, The State University of New York, Buffalo, New York 14260-3000, United States

**Keywords:** acyloin rearrangement, asymmetric synthesis, iminol rearrangement, ketol rearrangement, tandem reactions

## Abstract

In the presence of a suitable acid or base, α-hydroxyaldehydes, ketones, and imines can undergo isomerization that features the 1,2-shift of an alkyl or aryl group. In the process, the hydroxy group is converted to a carbonyl and the aldehyde/ketone or imine is converted to an alcohol or amine. Such α-ketol/α-iminol rearrangements are used in a wide variety of synthetic applications including asymmetric synthesis, tandem reactions, and the total synthesis and biosynthesis of natural products. This review explores the use of α-ketol rearrangements in these contexts over the past two decades.

## Introduction

**Figure 1:**
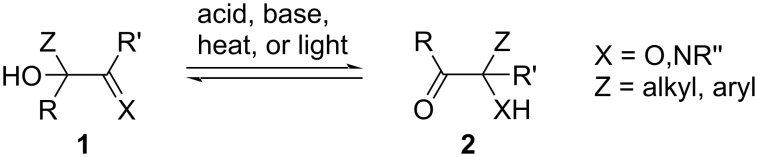
Generalized α-ketol or α-iminol rearrangement.

When treated with base, acid, heat, or light, many α-hydroxyaldehydes, ketones, or imines **1** undergo a 1,2-shift of one of the α-substituents to the adjacent unsaturated carbon, with a concomitant proton transfer to form compounds of type **2** (Figure 1) [1]. Differing from the related Wagner–Meerwein and pinacol/semipinacol rearrangements, the 1,2-shift does not require a leaving group or carbocation intermediate, as the neighboring π system is capable of accepting the migrating group. While the reaction is generally reversible, the product can be favored through four common strategies: (1) the use of aldehydes (R′ = H), which are usually less stable than their ketone counterparts; (2) ring expansion (Z, R = cyclic) or contraction (Z, R′ = cyclic) of strained cyclic α-ketols; (3) the use of α-dicarbonyl compounds (R′ = acyl, ester, amide, etc.), which lead to more stable β-dicarbonyl compounds; or (4) the use of imines (X = NR′′), which lead to more stable α-amino ketones.

The α-ketol or α-iminol rearrangement is a synthetic organic tool used for ring expansions and contractions and other isomerizations that is also used in some biological pathways [2–3]. Works featuring these reactions through mid-2002 have been thoroughly discussed in a past review by Paquette [1]. The current review expands on that work by providing an updated account from mid-2002 through early 2021, including the following recently developed applications: asymmetric synthesis, total synthesis, tandem reactions, and enzymatic rearrangements.

## Review

### Asymmetric α-ketol rearrangements

One major advancement in the field of α-ketol rearrangements is the development of methods for performing the reaction asymmetrically. This is possible by two approaches: (1) stereoselectively through the use of a chiral catalyst in the presence of a substrate possessing a prochiral migrating group or (2) stereospecifically by means of a chiral α-ketol.

**Figure 2:**
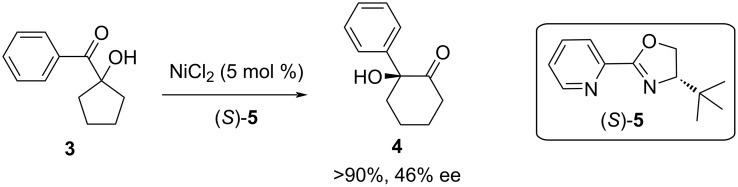
Nickel(II)-catalyzed enantioselective rearrangement of ketol **3** to form the ring-expanded and chiral product **4**.

As an example of an enantioselective rearrangement, complexes of nickel(II) with a series of chiral 1,2-diaminopropane or pyridineoxazoline ligands were evaluated for their conversion of **3** into the cyclohexanone product **4** (Figure 2) [4]. The best results were obtained with 2-[4-(*S*)-*tert*-butyloxazolin-2-yl]pyridine ((*S*)-**5**), which gave >90% yield of (*S*)-**4** in 46% ee.

**Figure 3:**
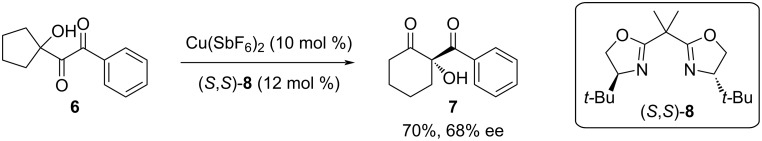
Enantioselective ring expansion of β-hydroxy-α-dicarbonyl **6** catalyzed by a chiral copper-bisoxazoline complex.

In a similar investigation except with copper(II) as the metal and β-hydroxy-α-diketone **6** as the substrate, the catalyst containing the chiral bisoxazoline ligand (*S*,*S*)-**8** led to the α-ketol rearrangement product **7** in 70% yield and 68% ee (Figure 3) [5]. The study further demonstrated the effectiveness of the catalyst for a series of analogues of **6** bearing various replacements for the phenyl group, often proceeding with greater than 80% ee.

**Figure 4:**
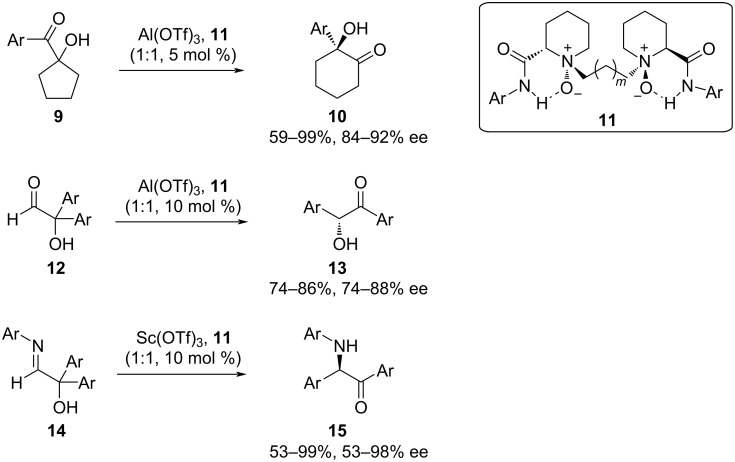
Enantioselective rearrangement of ketols **9** and **12** and hydroxyaldimine **14** catalyzed by Al(III) or Sc(III) liganded by **11**. Ligand **11**: for **9**, *m* = 1 and Ar = 2,6-iPr_2_C_6_H_3_; for **12**, *m* = 1 and Ar = 2,6-Me_2_C_6_H_3_; and for **14**, *m* = 0 and Ar = 2,4,6-iPr_3_C_6_H_2_.

As a third example of an enantioselective α-ketol rearrangement, Dai et al. used a chiral Al(III) catalyst to induce the rearrangement of **3** (Ar = Ph) and several aryl derivatives **9** (Figure 4) [6]. Among the *N*,*N*′-dioxide ligands explored, **11**, which was derived from ʟ-pipecolinic acid, was the most effective. Optimized conditions for substrate **3** (**11**, *m* = 1; Ar = 2,6-iPr_2_C_6_H_3_) led to yields approaching 99% with 91% ee. Addition of substituents on the phenyl group maintained excellent enantioselectivity, with 84% to 92% ee. Most of the twelve derivatives tested also had excellent yields (>85%), but the two most strongly electron-withdrawing, *para*-CF_3_ and *meta*-CH_3_, had lower yields (59% and 67%, respectively).

Instead of a ring expansion driving the rearrangement, the authors also tested a series of α,α-diaryl-α-hydroxyaldehydes **12** and their corresponding aldimines **14** (Figure 4). These reactions required small adjustments to ligand **11**, with *m* = 1 and Ar = 2,6-Me_2_C_6_H_3_ working best for **12** and *m* = 0 and Ar = 2,4,6-iPr_3_C_6_H_2_ best for **14**. Sc(III) proved to be superior for rearrangement of α-hydroxyaldimines. Under these conditions, products **13** were obtained in poor-to-moderate yields but with ≥74% ee, while the α-amino ketone products **15** could be obtained in nearly quantitative yields and variable ee, from 53% to 98% [6].

**Figure 5:**
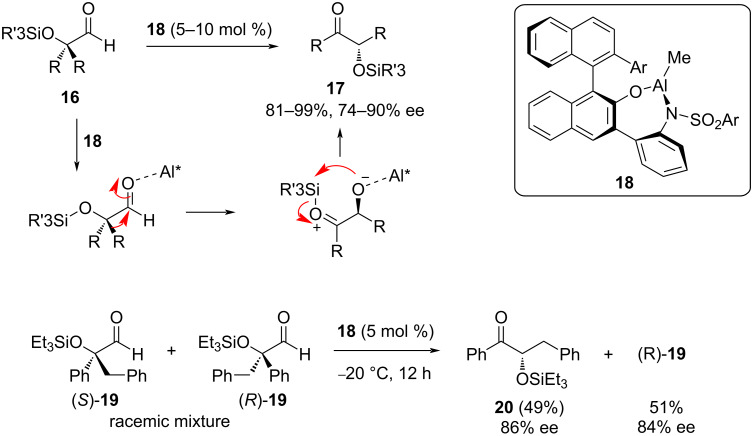
Asymmetric rearrangement of α,α-dialkyl-α-siloxyaldehydes **16** to α-siloxyketones **17** catalyzed by chiral organoaluminum Lewis acid **18**. The catalyst is also capable of kinetic resolution of a mixture of enantiomeric substrates. Al* = **18**.

A fifth example of an enantioselective α-ketol rearrangement provides a twist by demonstrating the ability to function on substrates protected as silyl ethers. Ooi et al. utilized an axially chiral organoaluminum Lewis acid catalyst (**18**) to convert a series of α,α-dialkyl-α-siloxyaldehydes **16** to α-siloxyketones **17** in high yields and >74% ee (Figure 5) [7]. This reaction is noteworthy for its tolerance of silyl protecting groups, which are transferred between the oxygen atoms after the alkyl migration, making it especially useful in synthetic strategies using these protected intermediates. Additionally, the reaction was found to be useful in the kinetic resolution of a racemic mixture of chiral substrates possessing both alkyl and aryl α-substituents. For example, only the *S* enantiomer from a racemic mixture of **19** was capable of being rearranged; (*R*)-**19** was recovered from the reaction mixture with 84% ee, whereas (*S*)-**19** had been stereospecifically converted to **20** in 49% yield and 86% ee (Figure 5).

**Figure 6:**
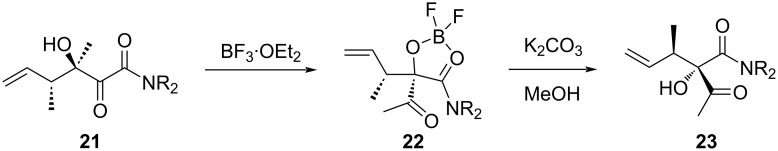
BF_3_-promoted diastereospecific rearrangement of α-ketol **21** to difluoroalkoxyborane **22**.

The final example of asymmetric α-ketol rearrangements utilizes asymmetric induction arising from the chiral alcohol. When the ketol’s carbonyl is located at the α position to an amide, as in β-hydroxy-α-ketoamide **21**, the difluoroalkoxyborane intermediate **22** that results from BF_3_-promoted α-ketol rearrangement can be isolated chromatographically. Subsequent methanolysis yields α-hydroxy-β-ketoamide **23** (Figure 6). The overall reaction occurs diastereospecifically, with greater than 80% yield across each of 5 distinct amide derivatives [8].

### Tandem α-ketol rearrangements

Because α-ketol rearrangements can be initiated by simple reagents like a Brønsted base or Lewis acid, the possibility exists to couple the rearrangement to other compatible reactions without any intervention. Such tandem reactions are attractive synthetic “tricks” that can allow for complex modifications with efficiency and often high selectivity. This short section introduces this concept by application to isolated reactions, while in the following section, examples of tandem reactions in total syntheses are given.

**Figure 7:**
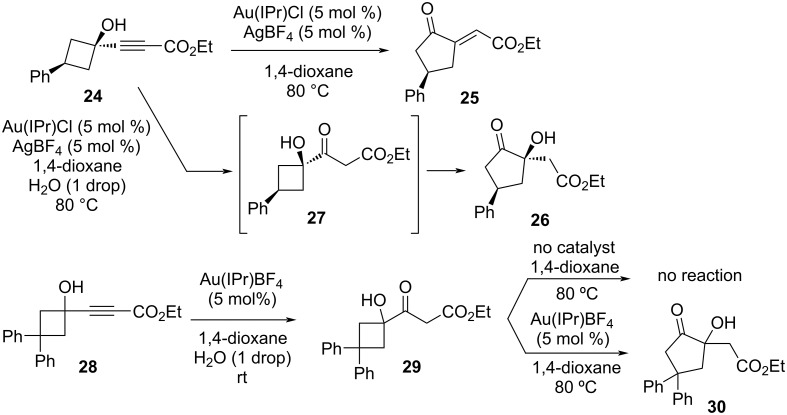
In the presence of a gold catalyst and water in 1,4-dioxane, 1-alkynylbutanol derivatives undergo tandem hydration and α-ketol rearrangement to give the corresponding ring-expanded cyclopentanones. IPr = *N*,*N*′-bis(2,6-diisopropylphenyl)imidazole-2-ylidene.

Inspired by the direct ring expansion of 1-alkynylcyclobutanols to α-methylenecyclopentanones catalyzed by gold reported by Toste and co-workers [9], Kim et al. hypothesized that by conducting the reaction in the presence of water, the reaction would be diverted to an initial hydration followed by an α-ketol rearrangement [10]. First, it was determined with model 1-alkynylcyclobutanol derivative **24** that in anhydrous conditions, the major product was **25**, resulting from the expected direct ring expansion (Figure 7). When the reaction was repeated in the presence of a single drop of water, however, only product **26** was observed, presumably by the hydration–rearrangement sequence. The intermediacy of the cyclobutyl α-ketol **27** was indirectly confirmed using diphenyl derivative **28**, which has a lower tendency for ring expansion. At room temperature, this substrate stopped at the hydrated intermediate **29**, which could be isolated and remained intact when heated to 80 °C alone in 1,4-dioxane. Inclusion of the gold catalyst while heating, however, initiated the rearrangement to **30**, confirming the importance of Au(I) in catalyzing both steps in the tandem reaction. Notably, this is the first time in the literature that α-ketol rearrangements have been initiated by a gold catalyst. Optimization revealed that the tandem reaction is best performed with Au(JohnPhos)SbF_6_ in 1,4-dioxane with a single drop of water.

### α-Ketol rearrangements implemented in total syntheses

**Figure 8:**
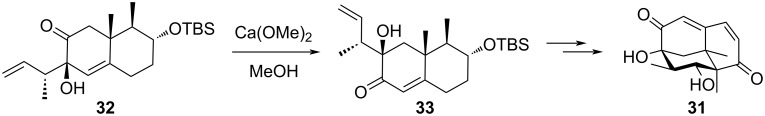
The diastereospecific α-ketol rearrangement of **32** to **33**, part of the total synthesis of periconianone A (**31**).

Although rare, α-ketol rearrangements have been included in the total syntheses of some natural products with high success. In the first total synthesis of periconianone A (**31**), an α-ketol rearrangement was used to shift a four-carbon chain one position on the bicyclic molecule **32** using the base calcium methoxide with a yield of 70% of **33** and a diastereomeric ratio of 3:1 (14:1 after separation) (Figure 8) [11]. Note that **33** bears an enone moiety, and therefore, this reaction is apparently the first example of the use of conjugation to drive an α-ketol rearrangement. This study elegantly illustrates the synthetic power of α-ketol rearrangements, whose suprafacial migration ensured that the α-ketol moiety present in the target product **31** was installed with correct stereochemistry. Taking inspiration from the above work from the Gademann group, Kalmode et al. incorporated the same synthetic strategy into their total synthesis of racemic periconianone A [12]. Interestingly, these authors not only tested the natural product for neural anti-inflammatory activity but also the two immediate synthetic precursors containing simpler decalin systems (identical to **32** and **33** but with an additional conjugated double bond) and found the α-ketol rearranged product superior to periconianone A.

**Figure 9:**
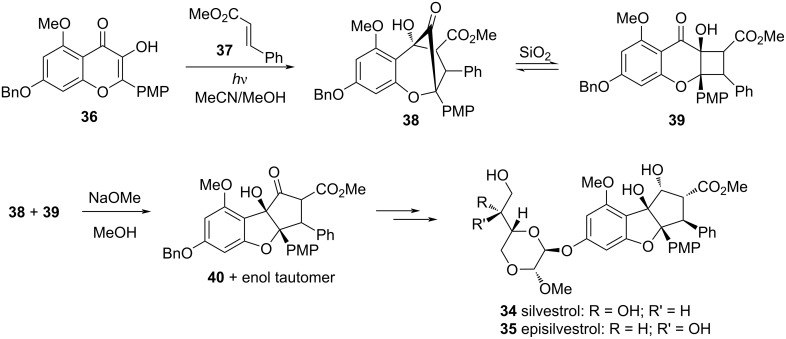
Two α-ketol rearrangements, one catalyzed by silica gel on **38** and the other by NaOMe on both **38** and **39**, part of the total synthesis of silvestrol (**34**) and episilvestrol (**35**). PMP = *p*-methoxyphenyl.

Another example of an α-ketol rearrangement in a total synthesis is in the preparation of silvestrol (**34**) and episilvestrol (**35**), natural products with potent anticancer properties. The step prior to the rearrangement involved a photoinduced [3 + 2] cycloaddition between hydroxyflavone **36** and methyl cinnamate (**37**), resulting in the bicyclic α-ketol **38** as a mixture of diastereomers (Ph and CO_2_Me groups trans) (Figure 9) [13]. Interestingly, when this mixture was subjected to silica gel chromatography, not only could the isomers of **38** not be separated, they also underwent silica-catalyzed α-ketol rearrangement to cyclobutane **39**. This rearrangement, however, was of no consequence because addition of sodium methoxide to the mixture of **38** and **39** induced a second α-ketol rearrangement to **40** as a tautomeric mixture. The same research group later utilized the same [3 + 2] cycloaddition and α-ketol rearrangement approach to prepare the 2′′′-epimer of **35**, which bears an inverted methyl acetal in the dioxane ring, but this compound was less active at inhibiting protein translation [14].

**Figure 10:**
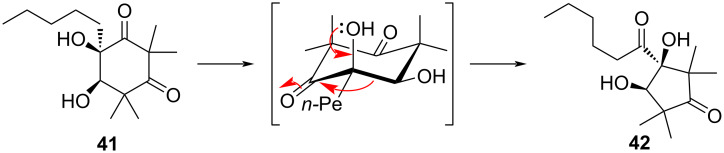
α-Ketol rearrangement of triumphalone (**41**) to isotriumphalone (**42**) via ring contraction.

Triumphalone (**41**) and isotriumphalone (**42**) represent a class of oxidized phloroglucinol natural products isolated from the Australian plant *Melaleuca triumphalis.* In their total synthesis of these two compounds in racemic form, Nishimura et al. discovered that **41** spontaneously undergoes an α-ketol rearrangement to form **42** in protic solvents (Figure 10) [15]. NMR solvent screening revealed that among CDCl_3_, DMSO-*d*_6_, C_6_D_6_, and CD_3_OD, **41** only isomerized at room temperature into **42** when dissolved in CD_3_OD (60% conversion over ≈5 days, as determined by ^1^H NMR). Additionally, purified **42** was not observed to undergo any α-ketol rearrangement reaction, although the authors did not discuss why the reverse reaction did not occur.

**Figure 11:**
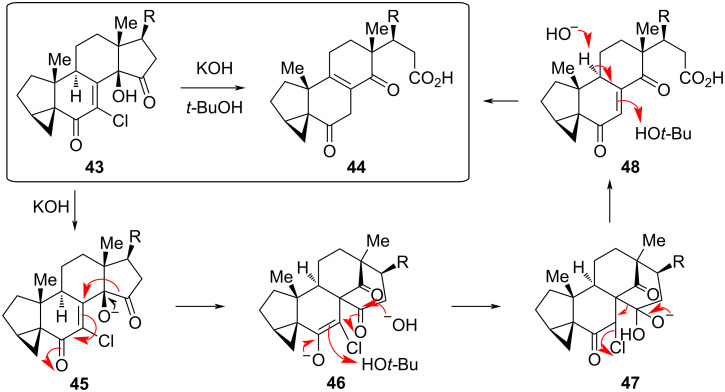
Tandem reaction of strophasterol A synthetic intermediate **43** to **44** through a vinylogous α-ketol rearrangement of **45** to **46**.

As was alluded to earlier, tandem reactions that include an α-ketol rearrangement have been incorporated into some total synthesis schemes. In the total synthesis of strophasterol A, a member of a structurally unprecedented class of secosterols, when intermediate **43** was treated with base, it was quantitatively converted to **44** (Figure 11) [2]. The proposed mechanism for the tandem reaction involves three hydroxide-promoted steps, beginning with a vinylogous α-ketol rearrangement to **46**. Following protonation of the enolate and addition of hydroxide to the carbonyl on the D ring, **47** rearranges with loss of chloride to give enedione **48**. Base-catalyzed isomerization yields **44**, which is apparently more stable despite the reduction in conjugation.

**Figure 12:**
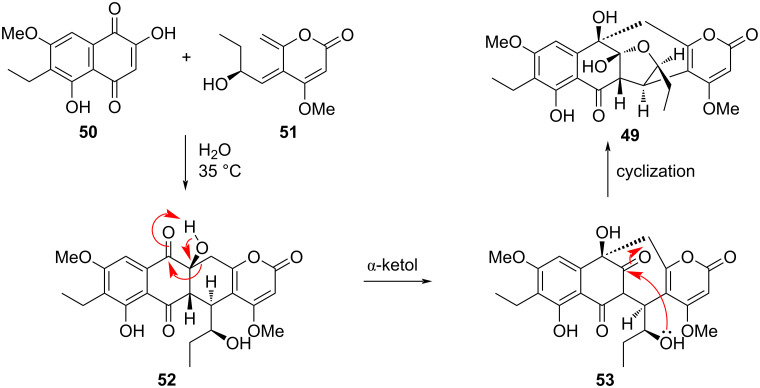
Tandem reaction consisting of a Diels–Alder cycloaddition followed by an α-ketol rearrangement, part of the total synthesis of delitschiapyrone A (**49**).

Another example of a tandem α-ketol rearrangement was used in the total synthesis of delitschiapyrone A (**49**), a cytotoxic natural product with previously demonstrated efficacy against several cancer cell lines. The final steps of the synthesis include a Diels–Alder reaction between **50** and **51**, an α-ketol rearrangement of **52**, and a hemiketal-forming cyclization of **53**, all of which occurred as a cascade (Figure 12) [16]. Over a series of optimizations, it was found that performing this cascade in a solution of water at 35 °C over the course of 2.5 days produced a 75% yield of **49** and a 22% yield of the Diels–Alder adduct, showing that the first step of the cascade had near-quantitative yield, diastereoselectively and regioselectively. The authors speculated that it is possible, considering their own reaction efficiency in conditions reminiscent of natural ones, that the biosynthesis of delitschiapyrone A could occur non-enzymatically in nature by a similar process.

**Figure 13:**
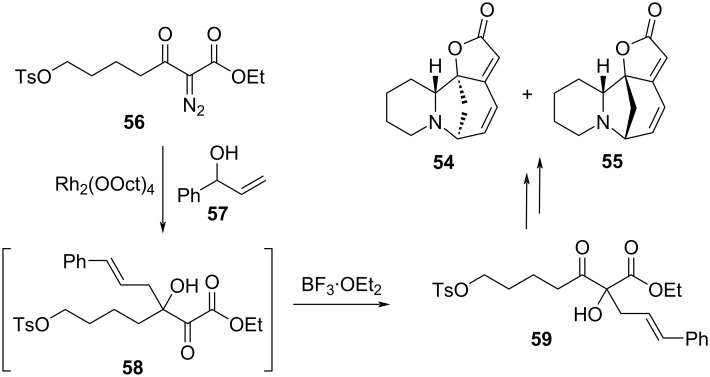
Single-pot reaction consisting of Claisen and α-ketol rearrangements, part of the total synthesis of (±)-securinine (**54**) and (±)-allosecurinine (**55**).

The final example of a tandem reaction involving an α-ketol rearrangement in a total synthesis was employed by Chen et al. in the preparation of (±)-securinine (**54**) and (±)-allosecurinine (**55**), biological alkaloids with diverse biological activities [17]. The key step first involved the rhodium carbenoid-mediated OH insertion/Claisen rearrangement following complexation of diazoester **56** and allylic alcohol **57** to produce intermediate **58** (Figure 13). Addition of BF_3_·OEt_2_ to the reaction mixture then catalyzed the α-ketol rearrangement to **59**. Note that this reaction took advantage of the thermodynamically preferred conversion of an α-ketoester to a β-ketoester seen in other examples in this review. Although not technically a tandem reaction due to the need to add a reagent to continue the cascade, the sequence of reactions nevertheless accomplishes the same goal of connecting two carbon-skeleton alterations conveniently in one pot with a combined yield of 45%.

### α-Ketol rearrangements in biosynthetic reactions

**Figure 14:**
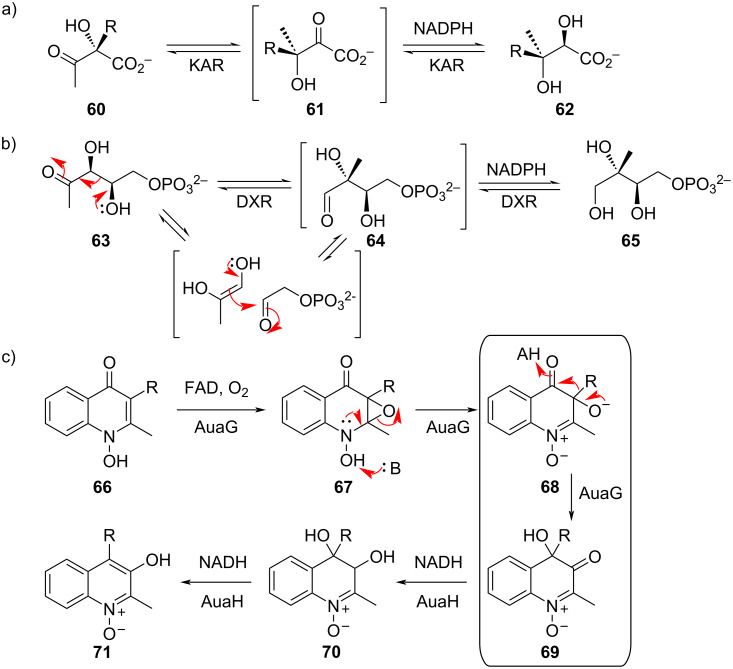
Enzyme-catalyzed α-ketol rearrangements. a) Ketol-acid reductoisomerase (KAR) catalyzes the rearrangement of (2*S*)-acetolactate (**65**, R = Me) or (2*S*)-acetohydroxybutyrate (**65**, R = Et) to **66**, followed by reduction by NADPH to **67**. b) Despite the similarity in reaction, 1-deoxy-ᴅ-xylulose-5-phosphate reductoisomerase (DXR) instead uses a retro-aldol/aldol sequence to accomplish its rearrangement of **68** to **69**. c) The secondary metabolite aurachin C (**71**) is oxidized by the FAD-dependent monooxygenase AuaG to epoxide **72**, which upon deprotonation by an enzymatic base (B), ring opens and subsequently rearranges to **74**. AuaH uses NADH to reduce the ketone to diol **75**, which undergoes dehydration to give aurachin B (**76**).

At the time of this review, only two enzymes have been identified that catalyze an α-ketol rearrangement as part of their mechanism. Ketol-acid reductoisomerase (KAR), which is involved in the biosynthesis of branched-chain amino acids, takes as its substrate either (2*S*)-acetolactate (**60**, R = Me), which is ultimately converted into valine or leucine, or (2*S*)-acetohydroxybutyrate (**60**, R = Et), which eventually becomes isoleucine (Figure 14a). The enzyme catalyzes two consecutive reactions: an α-ketol rearrangement to generate a 3-hydroxy-2-ketoacid intermediate **61**, followed by NADPH-dependent reduction to the dihydroxylated product **62** [18]. Interestingly, another reductoisomerase known as 1-deoxy-ᴅ-xylulose-5-phosphate reductoisomerase (DXR), uses a different mechanism to accomplish the carbon-skeleton rearrangement of its substrate **63** [19]; kinetic isotope effect experiments have excluded an α-ketol rearrangement and instead support a stepwise retro-aldol/aldol sequence for formation of intermediate **64** (Figure 14b) [20–21]. The other enzyme believed to catalyze an α-ketol rearrangement is AuaG, which is a monooxygenase that uses FAD and molecular oxygen to convert aurachin C (**66**) to **69** (Figure 14c) [22]. Subsequent reduction and dehydration by AuaH produces aurachin B (**71**).

While the above are the only identified enzymes that catalyze α-ketol rearrangements so far, there is little doubt additional examples are to be discovered. The remainder of this section provides cases of biological pathways and reactions that have been hypothesized to involve α-ketol rearrangements.

**Figure 15:**
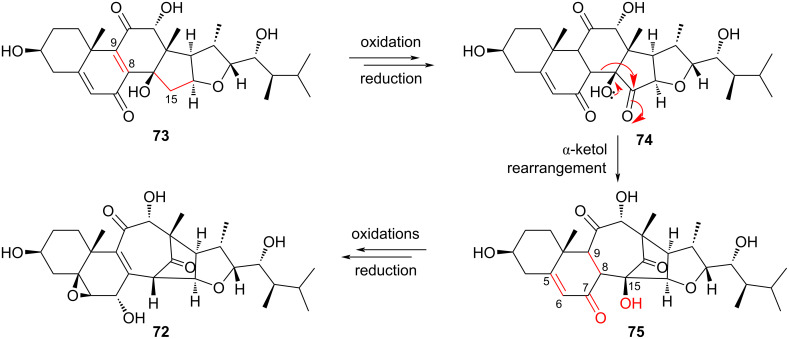
The conversion of asperfloroid (**73**) to asperflotone (**72**), featuring the ring-expanding α-ketol rearrangement of **74** to form **75**.

In a study proposing a biosynthetic pathway for the novel steroid asperflotone (**72**), it was suggested that its source was asperfloroid (**73**), a similar steroid isolated from the same source fungus, *Aspergillus flocculosus* [23]. First, reduction of the C8–C9 double bond and oxidation at C15 would provide α-ketol **74** (Figure 15). Next, ring-expanding rearrangement is proposed to form **75**. Finally, the C7 ketone is reduced, the C8–C9 bond is oxidized back to an alkene, the C5–C6 double bond is oxidized to an epoxide, and C15 is oxidized to a tertiary alcohol to yield **72**. The authors not only structurally characterized asperflotone and asperfloroid but also demonstrated their immunosuppressive activity against IL-6 production in induced THP-1 cells. Thus, as they noted, these two steroids may be attractive targets for total synthesis, perhaps incorporating the ring-expanding α-ketol rearrangement proposed to occur biologically.

**Figure 16:**
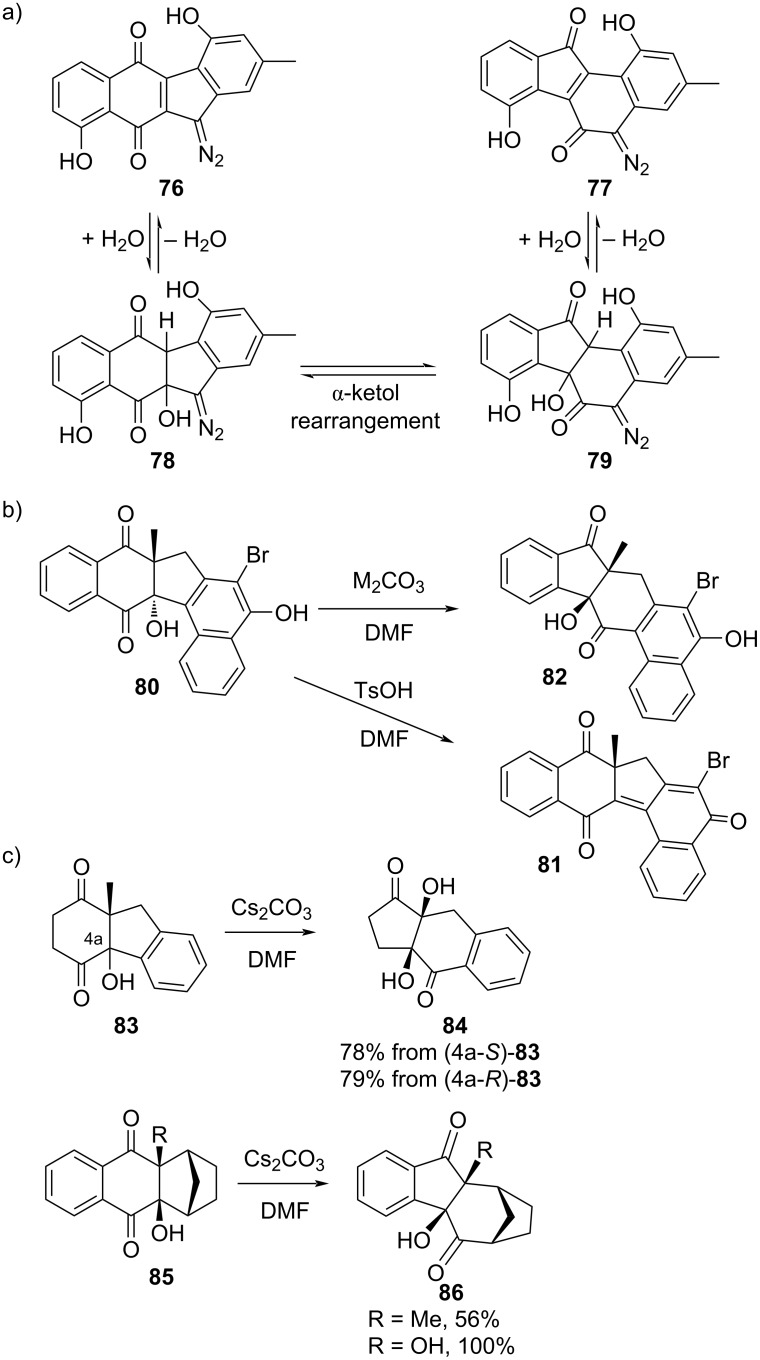
Hypothetical interconversion of natural products prekinamycin (**76**) and isoprekinamycin (**77**) and chemical models of their proposed α-ketol intermediates. a) The natural products are believed to interconvert biologically by a reversible sequence of hydration–rearrangement–dehydration. b) α-Ketol rearrangement of **80**, an analogue of proposed intermediate **78**, occurs with base (M = K or Cs), but dehydration to **81** occurs with acid. c) Substrate scope of similar Cs_2_CO_3_-catalyzed α-ketol rearrangements.

Prekinamycin (**76**) and isoprekinamycin (**77**) are diazo compounds isolated from *Streptomyces murayamensis* that differ in their ring system, with the former containing a fused 6-6-5-6 skeleton and the latter containing a 6-5-6-6 skeleton. Due to their similarity and common source, it had been hypothesized by several groups that one could be the biological precursor of the other by means of a reversible hydration–rearrangement–dehydration sequence (Figure 16a) [24–26]. The presence of the diazo group in intermediates **78** and **79** is noteworthy in that this functional group has not yet been demonstrated to be compatible with an α-ketol rearrangement. To test the feasibility of their hypothesis, Kawamura et al. subjected **80** to basic (Cs_2_CO_3_ or K_2_CO_3_) and acidic (TsOH) conditions [27]. While TsOH led only to dehydration product **81**, the bases exclusively generated **82** as the result of an α-ketol rearrangement (Figure 16b). While 6-6-5-6 ring systems were not explored, Cs_2_CO_3_ was tested on several other fused multi-ring α-ketols, leading to the expected rearranged products in moderate to quantitative yields in all cases where competing dehydration was not possible (Figure 16c). The authors concluded that further studies of this type of rearrangement in a biological system are currently being attempted.

**Figure 17:**
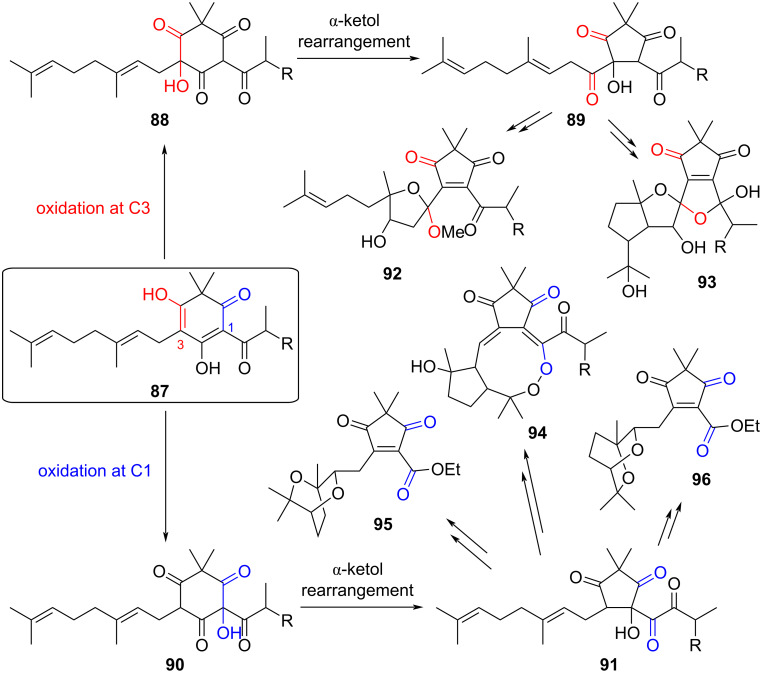
Proposed biosynthetic pathway converting acylphloroglucinol (**87**) to isolated elodeoidins A–H **92**–**96**. Oxidation at C3 followed by α-ketol rearrangement is believed to give rise to **92** and **93**, while a similar sequence at C1 is proposed to yield **94**–**96**. R = Me or Et.

As a final example of natural products believed to form as a result of an α-ketol rearrangement, Li et al. isolated and characterized eight novel acylphloroglucinol meroterpenoids, known as elodeoidins, from the herb *Hypericum elodeoides* [28]. The authors proposed that these molecules derived from common acylfilicinic acid precursors **87** through two distinct pathways based on the regiochemistry of an oxidation–α-ketol rearrangement sequence (Figure 17). If oxidation occurs at C3, then a subsequent ring-contracting α-ketol rearrangement would form **89**. From here, a series of oxidation, cyclization, methylation, and/or reduction steps yield **92** and **93** (each representing two isolated products, one with R = Me and the other with R = Et). Oxidation at C1 and α-ketol rearrangement to **91**, on the other hand, is proposed to give rise to isolated products **94** (R = Me, Et), **95**, and **96**. Interestingly, **94** showed anti-inflammatory activity.

### α‑Iminol rearrangements

Whereas α-ketol rearrangements must be driven thermodynamically by the presence of a destabilizing feature in the reactant (e.g., aldehyde, ring strain, or α-carbonyl group), α-iminols are typically less stable than their α-amino ketone products. In the presence of a Brønsted acid, protonation of the amine product can be used to drive the rearrangement to completion. Thus, favorable yields and stereoselectivities can be realized by first converting the α-ketol to an α-iminol by condensation with a suitable amine, provided that the amino group resulting from the rearrangement is compatible with the planned synthesis.

**Figure 18:**
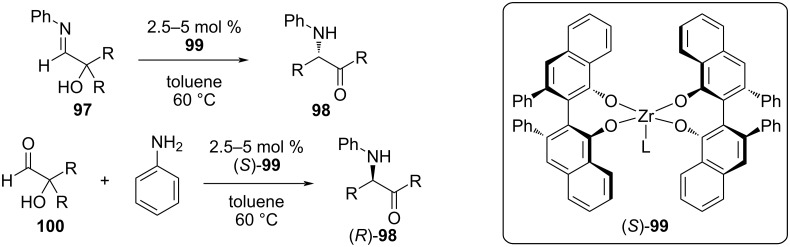
α-Iminol rearrangements catalyzed by VANOL Zr (**99**). The rearrangement can be conducted with preformed iminol **97** or from a mixture of aldehyde **100** and aniline.

In recent years, the Wulff group has made significant progress in the development of catalysts for asymmetric α‑iminol rearrangements. Inspired by the use of a BOROX catalyst in a series of asymmetric imine reactions, the group performed a catalyst screen for the conversion of **97** to **98** (Figure 18) [3]. Out of nearly twenty catalysts tested, (*R*)-VANOL Zr (**99**) produced the best results, with 5 mol % in toluene at 80 °C for 1 hour giving 96% yield of (*S*)-**98** with 97% ee. Impressively, raising the temperature to 160 °C for only 30 s gave 95% yield with 89% ee. The substrate scope revealed broad tolerance for aryl (except for *p*-trifluoromethyl) and aliphatic R groups, with yields and ee in excess of 90% in most cases.

**Figure 19:**
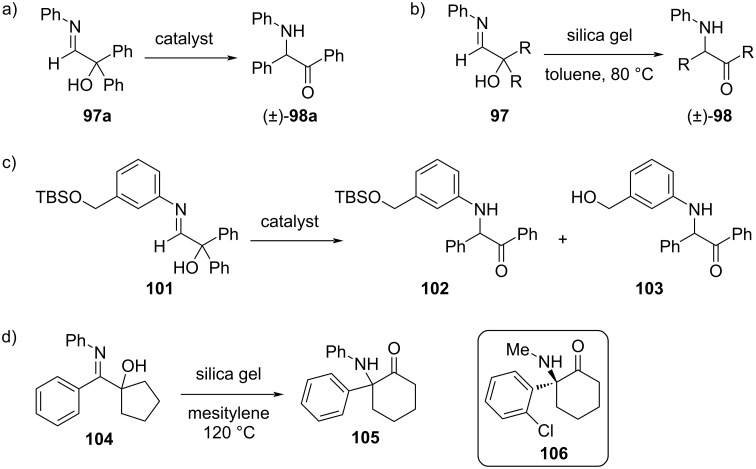
α-Iminol rearrangements catalyzed by silica gel and montmorillonite K 10. a) For **102a** (**102** with R = Ph), silica gel and montmorillonite K 10 gave ≥95% yields, while Brønsted acids, other Lewis acids, and NaOEt generally performed much more poorly. b) Electron-rich R groups rearrange efficiently in the presence of silica gel but not the electron-deficient *p*-trifluoromethylphenyl derivative. c) In the presence of silica gel or montmorillonite K 10, TBS-protected **101** is converted to **102** without any side products, but when sulfuric acid is used, only the deprotected product **103** is detected. d) Silica gel-catalyzed rearrangement of **104** provided **105**, an analogue of the anesthetic ketamine (**106**).

The Wulff group later explored alternative Brønsted and Lewis acids that could effectively catalyze the rearrangement of symmetric α‑hydroxy aldimines [29]. A catalyst screen was performed on the model substrate **97a** (**97** with R = Ph; Figure 19a) using three different Brønsted acids (acetic acid, sulfuric acid, and *p*-toluenesulfonic acid), five different Lewis acids (Zn(OTf)_2_, Cu(OTf)_2_, Sc(OTf)_2_, silica gel, and montmorillonite K 10), and one base (NaOEt). Of these, silica gel and montmorillonite K 10 had the best yields (95% and 100%, respectively). A substrate scope was then determined for **97** using aryl, *n*-hexyl, and cyclohexyl R groups in the presence of silica gel at 80 °C (Figure 19b). The rearrangements proceeded well with electron-rich aryl groups, regardless of the position of substitution, but the reaction was especially slow for the electron-withdrawing *para*-trifluoromethyl derivative.

A major advantage of the use of silica gel and montmorillonite K 10 as catalysts is that they function under neutral conditions, permitting the use of acid-sensitive groups, such as silyl ethers. For example, **101** was converted to **102** in 70% and 96% yield, respectively, with silica gel and montmorillonite K 10, but with sulfuric acid, no **102** was formed, only the free alcohol (**103**) in 56% yield (Figure 19c). As an extension of this method, the authors demonstrated the formation of **105**, an analogue of the anesthetic ketamine (**106**), in 83% yield using silica gel (Figure 19d) [29].

**Figure 20:**
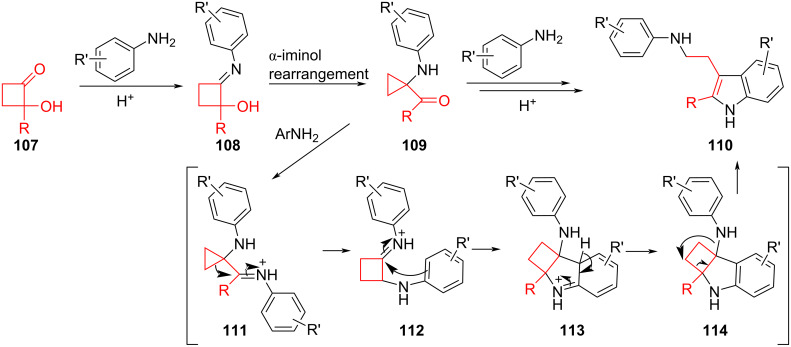
Synthesis of tryptamines **110** via a ring-contracting α‑iminol rearrangement. A mechanism for the final step has been proposed to occur through cyclobutyl intermediates **112** and **114** (some steps have been added/modified from the original source).

An α‑iminol rearrangement was utilized by Serusi et al. in a tandem reaction to synthesize functionalized tryptamines from 2-hydroxycyclobutanones **107** with a primary aniline [30]. Notably, the α‑iminol rearrangement results in a ring contraction to give the 2-aminocyclopropyl ketone intermediate **109**, which upon condensation with a second equivalent of aniline in the presence of a Brønsted acid undergoes a multistep rearrangement to form the indole group as part of the target tryptamine **110** (Figure 20). The one-pot conversions occurred successfully over a wide range of monosubstituted anilines, including various *para*-alkyl groups (65–72% yield), *para*-alkoxy and *para*-halogen substituents (45–69%), *ortho*-methyl (74%), and *meta*-methyl (80% as a 65:35 mixture of regioisomers). Electron-withdrawing groups (nitro, cyano, and methyl ester) stopped at the ketone intermediate.

**Figure 21:**
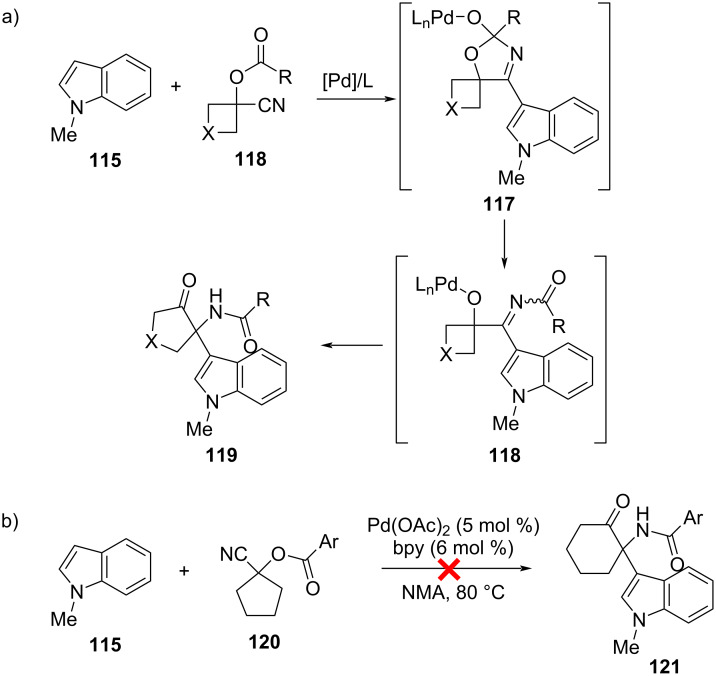
Tandem synthesis of functionalized α-amino cyclopentanones **119** from heteroarenes **115** and cyclobutanone-derived cyanohydrins **116**. a) Pd-catalyzed C–H addition of **115** to the nitrile group of **116** produces intermediates **117** and **118**. An α-iminol rearrangement with ring expansion subsequently produces **119**. b) Cyclopentanone-based substrates (**120**) do not react under the same conditions.

Interestingly, another tandem sequence that exploits the relief of ring strain from cyclobutane derivatives has been developed by Cheng et al. to prepare functionalized α-amino cyclopentanones [31]. In the presence of a palladium catalyst, an electron-rich heteroarene **115** first adds to the nitrile group in a 1-cyanocyclobutyl ester **116** to give a tetrahedral imine intermediate **117**. C–O bond cleavage produces an α-iminol intermediate **118**, which proceeds to rearrange by ring expansion to ultimately yield **119** (Figure 21a). Using the benzoate ester of **118** (R = Ph; X = CH_2_) as the substrate and *N*-methylindole as the heteroarene, optimal reaction conditions (95% yield) were found with Pd(OAc)_2_/bpy as the catalyst, *N*-methylacetamide as the solvent, and a temperature of 80 °C. Variously substituted indoles as well as esters of **118** (R = aryl, alkyl, vinyl) were generally well tolerated, but oxa- (X = O) and azo- (X = NR) cyclobutanes met with limited success. Alternative heteroarenes were much poorer, with pyrroles and thiophenes giving yields of ≈20% or less and benzofuran and benzothiophene failing to produce any product. Interestingly, a cyclopentanone-derived substrate (**120**) failed to yield the corresponding α-amino cyclohexanone **121** under the standard conditions used for cyclobutane derivatives (Figure 21b), highlighting the importance of the relief of ring strain in driving the rearrangement [31].

**Figure 22:**
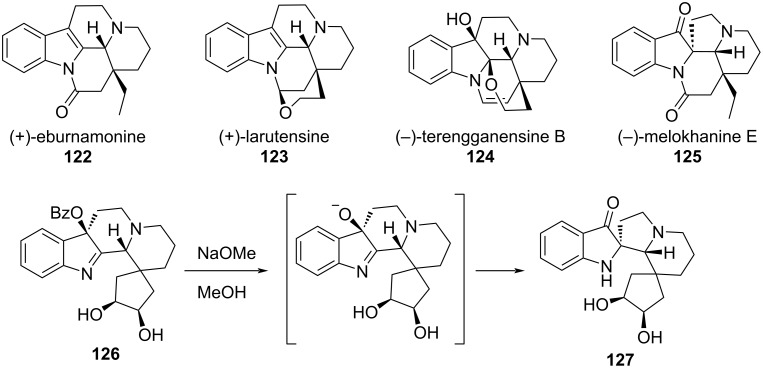
Four eburnane-type alkaloid natural products **122**–**125** were synthesized from common intermediate **127**, which was produced from known precursor **126** by an α-iminol rearrangement.

Like their α‑ketol counterparts, rearrangements of α‑iminols have also been used in total syntheses. In their total synthesis of four eburnane-type alkaloid natural products **122**–**125**, Li et al. utilized a ring-contracting α-iminol rearrangement to diastereoselectively install the spiroindolinone moiety that served as a key common intermediate (Figure 22) [32]. The rearrangement was triggered by saponification of the benzoyl ester in **126**, resulting in a 90% yield of **127**.

## Conclusion

Although α-ketol and α-iminol rearrangements are a somewhat specialized synthetic tool, they are steadily seeing increased use in organic chemistry. In particular, great progress has been made in asymmetric synthesis and tandem reactions, as illustrated in several examples in this review, and continued development in these areas can be expected in the coming years. It is becoming apparent that nature also utilizes these rearrangements in natural product biosynthesis, and one might expect to see an increase in the discovery of enzymes governing these reactions.
